# Worldwide distribution of human pythosis and biological characteristics of a *Pythium insidiosum* strain susceptible to antibiotics from China: a dual-scale study

**DOI:** 10.3389/fmed.2025.1629018

**Published:** 2025-12-09

**Authors:** Xiaoyun Liu, Qiuyue Diao, Mingliang Li, Yuting Yang, Jiayin Liang, Zehua Cui, Haiyan Zhang, Huiling He, Jiabao Huang, Hao Ren, Fengli Zhou, Tijiang Shan, Xiaopin Liao, Jian Sun, Kouxing Zhang

**Affiliations:** 1 Department of General Medicine of The Third Affiliated Hospital of Sun Yat-sen University, Guangzhou, China; 2 National Risk Assessment Laboratory for Antimicrobial Resistance of Animal Original Bacteria, South China Agricultural University, Guangzhou, Guangdong, China; 3 Department of General ICU of The Third Affiliated Hospital of Sun Yat-sen University, Guangzhou, China; 4 Department of Clinical Laboratory of The Third Affiliated Hospital of Sun Yat-sen University, Guangzhou, China; 5 College of Forestry and Landscape Architecture, South China Agricultural University, Guangzhou, China

**Keywords:** oomycetes, pythiosis, *Pythium insidiosum*, epidemiology, identification, antimicrobial treatment

## Abstract

**Introduction:**

Pythiosis, caused by *Pythium insidiosum* (*P. insidiosum*), is an emerging disease with high mortality and morbidity. Despite its clinical severity and geographical strain variations, diagnostic and treatment challenges persist.

**Materials and methods:**

We queried PubMed and Google Scholar for “*P. insidiosum*” and “pythiosis,” 1084 human pythiosis cases to generate a corresponding distribution map. Molecular biology, morphology, modified sporulation technique, and microscopic observation were employed to understand the biological properties of *P. insidiosum* with a strain of *P. insidiosum* isolated from a patient in Guangzhou. Drug susceptibility studies on the isolate were conducted both *in vitro* and *in vivo*.

**Results:**

Molecular biology, morphology, and biological processes confirmed that this strain was *P. insidiosum*. Epidemiological investigations have revealed that India and Thailand are hotspots for human pythiosis, and sporadic cases are increasing in China, the Americas, and Europe. The modified method of zoospore induction achieved 250-fold greater than the traditional method within 24 h. *In vitro* drug testing demonstrated superior antibiotic sensitivity (doxycycline (DOX) MIC 4 μg/mL; azithromycin (AZM) MIC 8 μg/mL) versus antifungals (> 128 μg/mL). Subcutaneous infection models in immunocompromised mice showed 80 and 90% survival with oral AZM/DOX monotherapy versus 20% in controls (*P* < 0.05), correlating with reduced hepatic fungal burdens and attenuated neutrophilic periarteritis.

**Conclusion:**

The distribution map underscores pythiosis as a growing climate-sensitive disease that requires enhanced surveillance in non-endemic regions. The modified method of zoospore induction and microstructure observation with Transmission Electron Microscopy (TEM) may be helpful for the rapid and sensitive detection of *P. insidiosum*. We also provided theoretical and technical support for the effective treatment of pythiosis in humans and animals with DOX and AZM in China, which offers novel insights into the potential of certain antibiotics as effective treatments.

## 1 Introduction

*Pythium insidiosum* (*P. insidiosum*), an oomycete pathogen of the *Stramenopila* lineage, was previously misclassified under the “fungus-like” category (1). *P. insidiosum* is not the only pseudofungal pathogen responsible for neglected infectious diseases. Organisms such as *Prototheca* spp. and *Rhinosporidium seeberi* also face diagnostic and therapeutic challenges due to their atypical biological nature and limited awareness among clinicians (2, 3). Additionally, other oomycetes, such as *Lagenidium* spp., have been increasingly recognized as emerging pathogens in animals and humans (4). Therefore, fungus-like pathogens require more attention from both clinicians and researchers.

As the sole Pythium species causing mammalian pythiosis, it is widespread in temperate, tropical, and subtropical areas and is a major ecological species in swamps (5–7). During the biphasic invasion cycle of *P. insidiosum*, motile zoospores, the primary infectious propagules, differentiate from sporangia and germinate into host-penetrating hyphae that establish tissue infection (8). *P. insidiosum* differs from true fungi in cell wall composition (lack of chitin but is rich in cellulose and β-glucans), mitochondrial structure, actin cytoskeleton, protein repertoire, incomplete sterol biosynthesis pathway, 70s ribosome and biflagellate zoospores that can actively swim in water for 20–30 min (1, 9). Human pythiosis presents in three major clinical forms: vascular, cutaneous/subcutaneous, and ocular (10). Animal pythiosis typically manifests as cutaneous proliferative, ulcerative nodules, or tumor-like masses on the limbs, scrotum, or abdomen, and gastrointestinal forms produce weight loss, vomiting, and segmental granulomatous enteritis (11). The diagnosis of pythiosis remains challenging and requires diligent records of medical history, physical examination of the patient, and laboratory information, including direct microscopic observation, culture identification and zoospore induction, serum *P. insidiosum*-specific antibodies, histological examination, and molecular analysis (10, 12). Differential diagnosis of pythiosis from other fungal infections, such as mucormycosis and entomophthoramycosis, is crucial. Mucormycosis is characterized by rapid tissue necrosis and angioinvasion, leading to thrombosis and infarction of the affected tissue. Entomophthoramycosis often presents with chronic indolent lesions and a granulomatous inflammatory response (13). Laboratory methods are helpful for accurate diagnosis. Motile zoospores in water can distinguish pythiosis from other fungi (14). Molecular methods, such as PCR assays, can also be used to differentiate *P. insidiosum* from other pathogens (15, 16).

Pythiosis burden analyses reveal a strikingly conflicting reality: 79.2% of affected animals were in America and Brazil, whereas 94.3% of human cases were in India and Thailand (17). The geographic distribution of this disease is increasing and poses a significant public healthh threat with high morbidity (vascular: 78%, ocular: 79%) and mortality (disseminated: 100%, vascular: 40%) (6). Pythiosis is commonly treated with surgical resection, immunotherapy, and antibacterial drugs (18, 19). Most treatments for pythiosis are surgical, although this course of action can considerably increase the economic burden, postoperative complications, and uncontrolled infections. The therapeutic properties of *P. insidiosum* antigens need to be improved for better prognosis in patients with pythiosis (19). Antimicrobial medication therapy achieves therapeutic efficacy by eliminating invasive microorganisms while minimizing costs and adverse effects. The repurposing of existing drugs is a safe and efficient strategy for the anti-*P. insidiosum* (20). However, the results of antimicrobials against *P. insidiosum* are inconsistent *in vitro* and *in vivo*, and no standardized antimicrobial susceptibility testing (AST) framework exists for this World Health Organization-listed priority oomycete. Therefore, understanding the geographic distribution of *P. insidiosum* is critical for developing targeted prevention strategies and optimizing the clinical management of pythiosis, particularly in regions experiencing a progressive rise in case incidence, such as China.

To comprehend the biological properties of *P. insidiosum* and make the effort to overcome this difficult clinical challenge, we observed the transition of *P. insidiosum* (*GZ2020*) (16) from hyphae to spore form using a microscope, and we used a modified sporulation technique to produce zoospores more quickly and plentifully. Importantly, we conducted drug sensitivity tests *in vitro* and *in vivo* on infected mouse models, which are sensitive to some antibacterial drugs. Our results will help repurpose existing drugs as a safe and efficient strategy against *P. insidiosum*.

## 2 Materials and methods

### 2.1 Study design

We isolated a strain of *P. insidiosum* (*GZ2020*) from a patient with skin ulcers. This pathogen was identified as *P. insidiosum* based on mycelial morphology on different media and molecular biology. We constructed a phylogenetic tree based on the genes to determine their evolutionary relationship. We observed its biological processes using scanning electron microscopy (SEM) and its inner structure using transmission electron microscopy (TEM). Additionally, we mapped the distribution of human pythiosis using the data from PubMed and Google Scholar and visualized it with R (4.4.1). We improved the sporulation induction method for *P. insidiosum*. We performed drug susceptibility tests with antibacterial and antifungal agents *in vitro* and drug susceptibility tests *in vivo* using a mouse model. The study design is shown in Supplementary Figure 1.

### 2.2 Epidemiological distribution

We searched the keywords “*P. insidiosum*” and “pythiosis” in PubMed^1^ (accessed date: 8 March 2025) and Google Scholar^2^ (accessed date: 11 March 2025). The clinical data of human pythiosis cases, including publication year, number of cases, and country of origin, were extracted and collected from the obtained literature. All collected cases met at least one of the following diagnostic criteria: (i) microbiological methods (such as culture identification, zoospore induction, and microscopic examination), (ii) histological assessment (histological examination and staining), and (iii) serological tests. (iv) Molecular assays. A total of 120 articles and 1314 cases were included. Potentially duplicated pythiosis patients were excluded from the study to prevent overestimation of the overall case number. The main criteria for considering a case as a duplicate were an identical institution, overlapping study period, and identical disease manifestation. 86 studies, including 1084 cases (Supplementary Data Sheet 1), were enrolled in the epidemiological distribution map. One case potentially infected in Brazil or Colombia was attributed to Colombia in this analysis.

### 2.3 Isolation and identification

The *P. insidiosum* (*GZ2020*) was isolated from a skin infection patient from the Third Affiliated Hospital of Sun Yat-sen University. We placed infected tissues on blood agar plates or 2% Sabouraud Dextrose Agar (Guangdong Huankai Microbial Technology Co., Ltd. Guangzhou, China) and incubated at 37 °C for 2 days. After hyphae growth, the culture was purified by passaging using dot-planting. According to previous studies, hyphae were picked and stained with lactophenol cotton blue (21, 22).

Genomic DNA from mycelia was extracted with a DNA Extraction Kit (Shanghai Shenggong Biological Engineering Co., Ltd. Shanghai, China), and gDNA amplification was performed using primers for different genes (15). We amplified gene LSU and COI as described by Robideau et al. (12) and Bala et al. (23) with only minor modifications: the reaction volume increased to 50 μL, the denaturation shortened to 2 min and the extension time lengthened to 80s for LSU and the extension time lengthened to 70s for COI. The polymerase chain reaction (PCR) products were separated by 1% agarose gel electrophoresis and stained for observation.

We spliced the sequences into complete sequences using DNAMAN (version 9.0, United States) and compared the amplified primer sequences with BLAST software on the NCBI website. Homology searches were performed in the GenBank database and compared with the identified *P. insidiosum* sequences.^3^ We utilized MAFTT (Version 7.0, JPN) to download sequences with high similarity and similar genera. We then used MEGA (Version 12.0, United States) with the maximum likelihood method to construct a phylogenetic tree to determine whether they were in the same branch as *P. insidiosum*.

### 2.4 Zoospore induction

**FIGURE 1:**
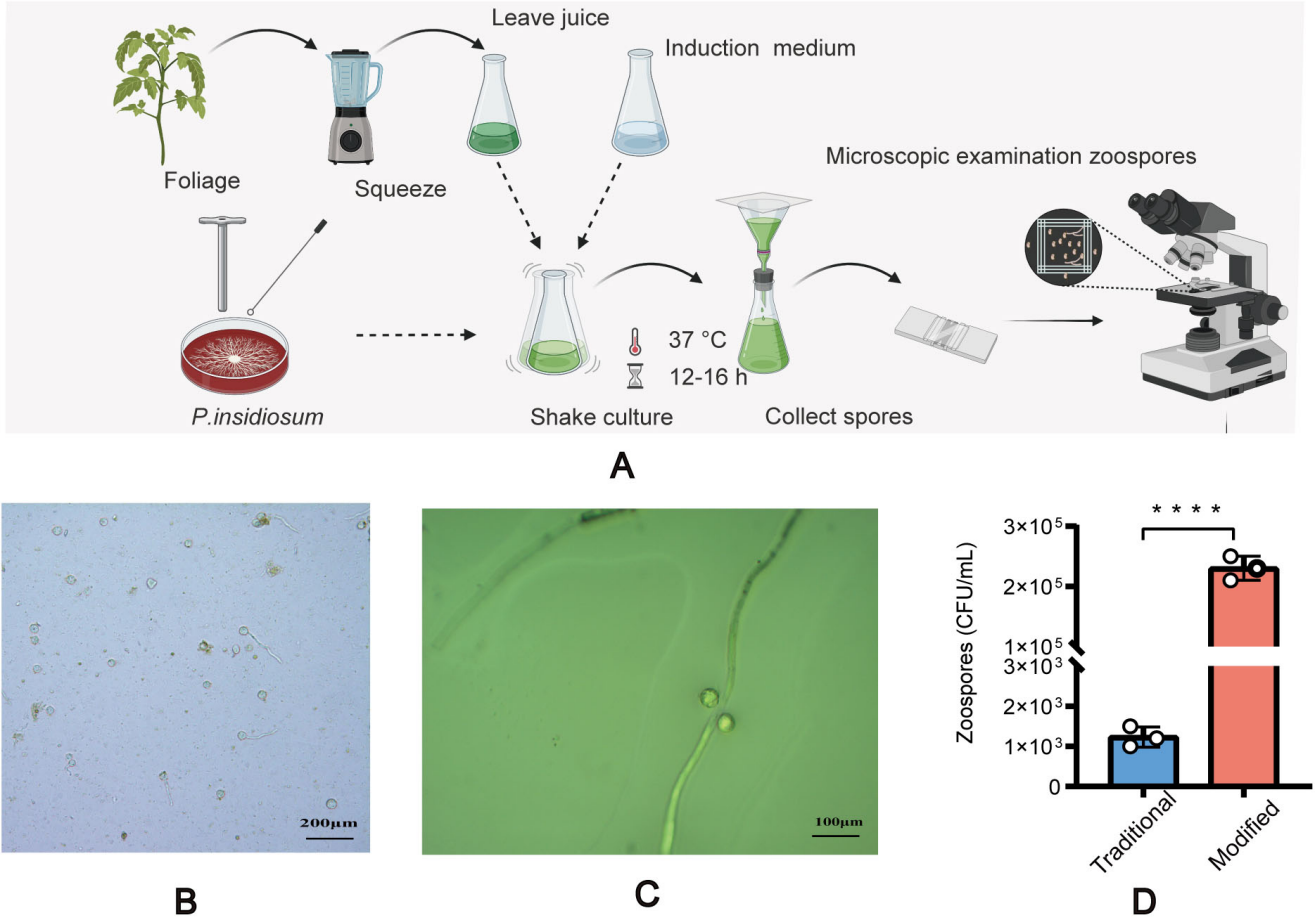
Induction of zoospore analysis. The step-by-step process of inducing spore formation **(A)**, Microscopic images of the sporulation of modified method **(B)**. Microscopic images of the sporulation using the traditional method **(C)**. A comparative graph of spore yield between the traditional method and the modified method **(D)**. All data are presented as mean ± SD, and the significances were determined by one-way ANOVA (****P < 0.0001).

This study used a modified method to induce sporulation (Figure 1A) in *P. insidiosum*, which requires an induction solution combined with plant sap. Liquid A included K_2_HPO_4_ ⋅ 3H_2_O (Tianjin Yongda Chemical Reagent Co., Ltd. Tianjin, China)11.4 g, KH_2_PO_4_ (Tianjin Yongda Chemical Reagent Co., Tianjin, China) 6.8 g, (NH_4_)_2_HPO_4_ (Tianjin Yongda Chemical Reagent Co Tianjin, China) 6.6 g, distilled water 50 mL, Liquid B includes MgCI_2_ ⋅ 6H_2_O (Tianjin Yongda Chemical Reagent Co., Ltd. Tianjin, China) 2.54 g, CaCl_2_ ⋅ 2H_2_O (Tianjin Yongda Chemical Reagent Co., Ltd. Tianjin, China)1.84 g, and distilled water 25 mL. Induction solution: Mix 0.5 mL of Liquid A and 0. 1 mL of Liquid B in 1,000 mL of distilled water, adjusted pH range 7.0 ∼ 7.1, stored at 4 °*C* (24). Extraction of plant sap: Annona squamosa L leaves (100 g) were weighed, distilled water (500 mL) was added, and sap was extracted, sterilized, and stored at 4 °C. Agar plugs with hyphae on the edge of fresh blood agar plates were inserted into 2% Sabouraud Dextrose Agar plates and incubated at 37 °C for 1 week. The agar plugs with mycelium were placed in centrifuge tubes with 2% Sabouraud Dextrose Broth (Guangdong Huankai Microbial Technology Co., Ltd. Guangzhou, China) culture solution and incubated at 37 °C and 180 rpm for 4 h. The broth was removed from the centrifuge tubes, and an equal amount of distilled water was added and incubated at 37 °C and 180 rpm for 1 h. The centrifuge tubes were incubated with an induction solution and plant sap at a volume ratio of 1:1 at 37 °C and 180 rpm for 10–24 h. We observed the production of zoospores and the release of zoospores from sporangia under a light microscope (MI52-N, Guangzhou Mingmei Optoelectronics Technology Co., Ltd., Guangzhou, China). We also compared the zoospore yield between our modified method and the traditional method (24). We used a Neubauer chamber to count the zoospores after zoosporogenesis (25, 26). We counted the zoospores on the leaves using a light microscope according to the traditional method.

### 2.5 Observation with SEM and TEM

Oomycete preparations were used to remove *P. insidiosum* from the induction solution. We soaked the samples in 1 mL of 2.5% glutaraldehyde fixative and rinsed them with 0. 1 mL phosphate buffer, washed them in 0. 1 mL phosphate buffer three times and fixed them in 1% osmium solution. After drying the samples with ethanol and dehydrating them (30,50,70,80, and 90% once for 10 min per concentration, and 100% 2 times for 10 min), the *developmental cycle of P. insidiosum* was observed using SEM (Verios 460, Field Electron and Ion Company (FEI), Brno, Czech Republic).

To evaluate the ultrastructural features of the hyphae, we applied an oomycete preparation to remove the hyphae from the induction solution. Samples were placed in 1 mL of a fixative solution containing 2.5% glutaraldehyde and 2% paraformaldehyde (2.5% GA + 2% PFA). The samples were then rinsed with 0.1 mL of phosphate buffer three times and fixed with a solution of osmium (1% osmium + 1% potassium ferricyanide, prepared at 0.2 M Morphotropic Phase Boundary). Subsequently, the samples were stained with uranyl acetate, dehydrated with ethanol (30,50,70,80, and 90% once for 15 min per concentration, and 100% 2 times for 20 min), washed with 100% acetone 2 times for 10 min, and infiltrated with a mixture of acetone and embedding agent (resin) (volume ration were 3:1,1:1 and1:3). Finally, the samples were polymerized for 24 h at 70 °C (27). 70–100 nm thick were obtained using an ultrathin sectioning machine. The sections were then placed on a nickel grid and stained with uranyl acetate and lead citrate. The internal morphology of the hyphae was examined using TEM (Talos F200S, FEI, Brno, Czech Republic) on a nickel grid.

### 2.6 Drug sensitivity *in vitro*

The susceptibility of *P. insidiosum* was tested using the broth microdilution method, following the modified Clinical and Laboratory Standards Institute (CLSI) M38-A2 protocol (28). In our study, zoospores were used for the drug susceptibility assays. The zoospores were resuspended in RPMI-1640 (Thermo Fisher Scientific, Waltham, United States) broth with a pH of 6.9–7.1, supplemented with 0.164 mL of 3-[N-morpholino] propane sulfonic acid (Guangzhou Xinglang Biotechnology Co., Ltd., Guangzhou, China), to achieve a final concentration of 2–3 × 10^∧^3 cells/mL (29). Microdilution 96-well plates were utilized to test the zoospore suspension (inoculum) against either RPMI-1640 alone (as a no-drug control) or various drug concentrations. After 24 h of incubation at 37 °C, the minimum inhibitory concentration (MIC) was determined according to other studies (30). We conducted three biological replicates to assess the sensitivity of *P. insidiosum* to several antibiotics (doxycycline (DOX), tetracycline (TET), erythromycin (ERY), and azithromycin (AZM]) (Shanghai Yuanye Biotech Co., Ltd., Shanghai, China), tigecycline (TIG) (Guangzhou Zhuorui Biotech Co., Ltd., Guangzhou, China), chloramphenicol (CHL), and linezolid (LZD) (Shanghai Maclin Biochemical Technology Co., Ltd., Shanghai, China), gentamicin (GCN) (Beijing Pubuxin Biotech Co. Ltd., Beijing, China), and florfenicol (FFC)(Guangzhou Weigu Technology Co., Ltd., Guangzhou, China), compound sulfamethoxazole (SMZ/TMP) (Shandong Fangming Pharmaceutical Group Co. Ltd., Shangdong, China) and antifungals (fluorocytosine and itraconazole [(Shanghai Yuanye Biotech Co., Ltd., Shanghai, China), amphotericin B (Shanghai Aladdin Biochemical Technology Co., Ltd., Shanghai, China), and fluconazole (Guangzhou Zhuorui Biotech Co., Guangzhou, China)].

### 2.7 Animal model and drug sensitivity *in vivo*

**FIGURE 2:**
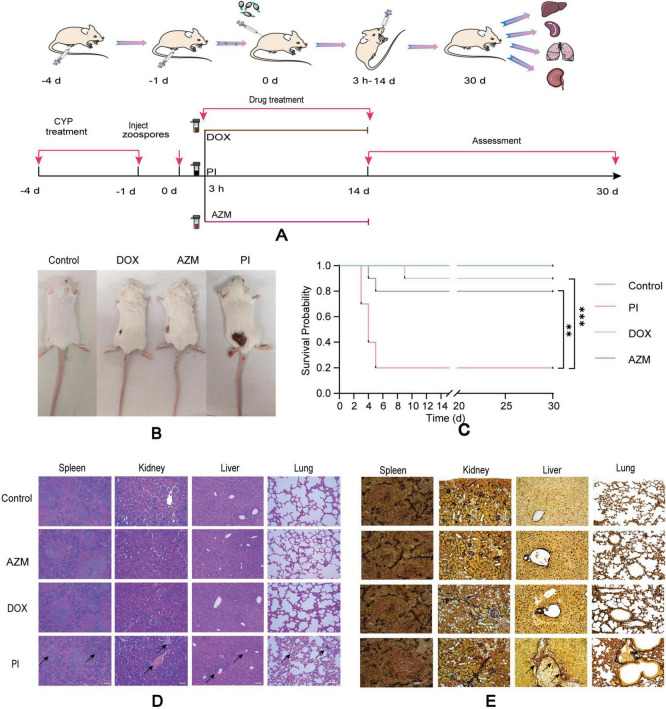
Schematic representation of drug treatment for infected pythiosis animals **(A)**. Changes in infected wounds in animals of different treatment groups **(B)**. Survival of mice after treatment with control, AZM, DOX, and PI **(C)**, (*n* = 10). Results of H&E staining of the liver, lung, spleen, and kidney in different treatment groups **(D)**. Results of tissue loading capacity tests using GMS staining on the kidneys, spleen, liver, and lungs of mice in different groups of treatment **(E)**. Scale bar: 100 μm. All data are presented as mean ± SD, and significance was determined by one-way ANOVA (**P < 0.01, ***P < 0.001). Control: immunosuppressed only (cyclophosphamide), uninfected; AZW, azithromycin; DOX, doxycycline; PI, saline.

This study utilized 7-week-old female BALB/c mice (18–22 g) that were pathogen-free and obtained from the Guangdong Medical Lab Animal Center in Guangzhou, China. The animal experimental procedures were conducted following the guidelines of the South China Agricultural University (SCAU) Institutional Laboratory Animal Care and Use and were approved by the SCAU Institutional Ethics Committee (2020C407). To induce neutropenia, two intraperitoneal injections of cyclophosphamide were administered at doses of 150 mg/kg on 4 days before infection and 100 mg/kg on 1 day before infection. On day 0, the mice were anesthetized using an intraperitoneal injection of ketamine-xylazine (Syntec Brasil Ltd., São Paulo, Brazil) at 50 and 10 mg/kg, respectively, respectively. Uninfected, just administered cyclophosphamide and completely untreated cohorts (*n* = 10) served as the baseline controls [immunosuppressed only, uninfected group (control group)]. Subsequently, subcutaneous injections infected mice with 2 × 10∧4 zoospores/mouse (31). The infected mice were randomly divided into three groups, each comprising 10 animals. From day 0 (3 h after zoospore inoculation) to day 14 after infection, the mice received one of the following treatments: (i) oral Saline (PI group), (ii) oral AZM (50 mg/kg of body weight every 12 h; AZM group), (iii) oral DOX (40 mg/kg of body weight every 12 h; DOX group). The experimental process is shown in Figure 2A. Throughout the study, the animals were monitored 3–4 times daily for clinical signs such as changes in breathing, weight, mobility disorders, etc. Criteria for euthanizing the animals during the study included physical and mental alertness, chronic diarrhea, and bleeding. At the end of the experiment, all surviving animals were euthanized by deepening the anesthesia using thiopental (60 mg/kg). Histopathological analyses were performed. The number of hyphae in the tissue slices was measured using Gomori’s methenamine silver (GMS) staining, which enabled the observation of *P. insidiosum* in the organs. Hematoxylin and eosin (H&E) staining was used to identify inflammatory responses.

### 2.8 Statistical analysis

Results were presented as means ± standard deviation (SD). The statistical analysis was performed with GraphPad Prism (Version 8, United States). Unless otherwise specified, we used the unpaired *t*-test or one-way ANOVA to assess the statistical significance of comparisons (*P < 0.05, **P < 0.01, ***P < 0.001, ****P < 0.0001, ns: not significant).

## 3 Results

### 3.1 Epidemiological distribution of human pythiosis cases

**FIGURE 3:**
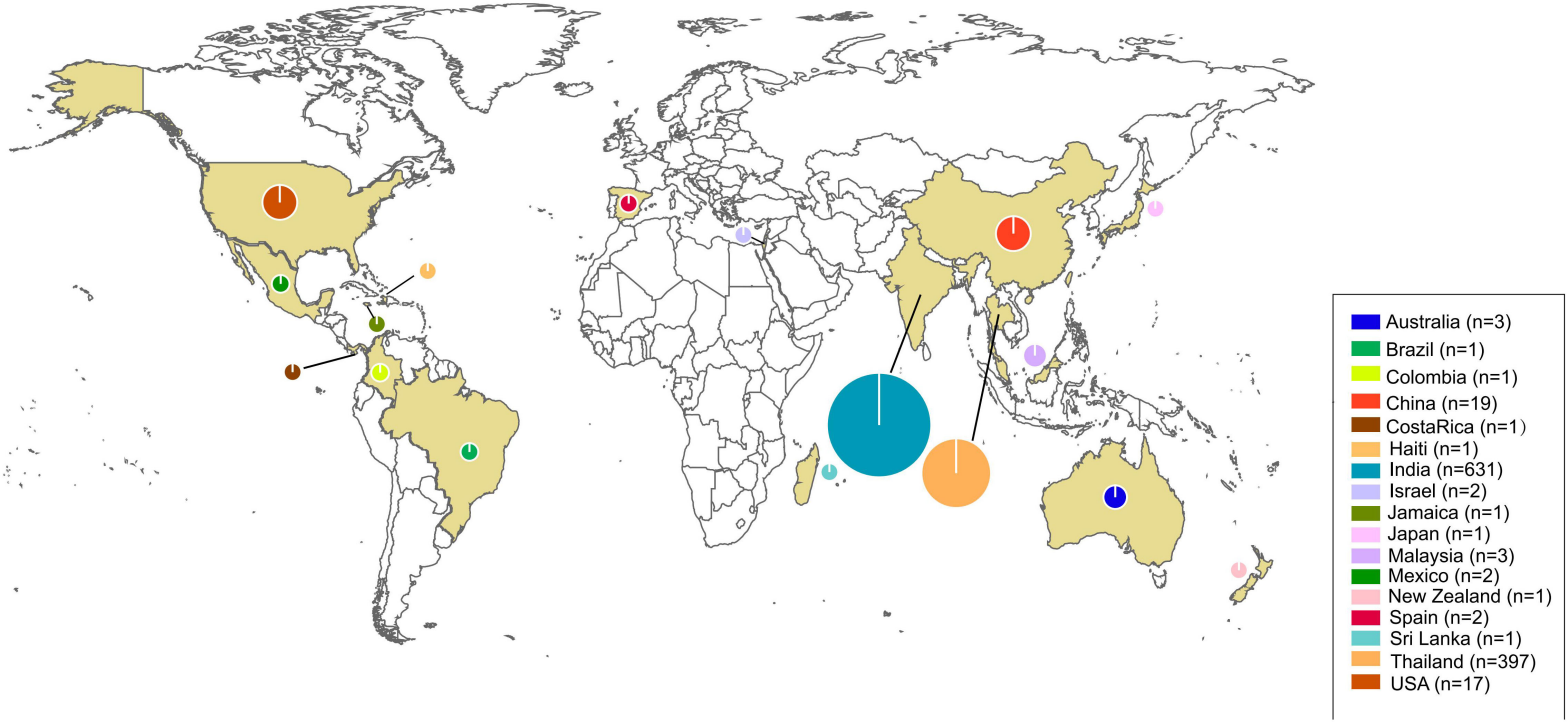
Distribution world map of human pythiosis. The size of the circles represents the number of cases in each region and yellow areas were the regions with reported cases. The legend in the lower right corner indicates the specific case counts.

Seventeen countries reported human pythiosis cases. The epidemiological distribution map revealed that human pythiosis cases maintained their predominant coastal distribution pattern across Asia and the Americas, with India and Thailand continuing to demonstrate the highest endemic burden (94.83% of total reported cases). The secondary prevalence clusters were China (*n* = 19) and the United States (*n* = 17), representing 1.75 and 1.57% of the global case load, respectively (Figure 3).

### 3.2 Isolation and identification of *P. insidiosum*

**FIGURE 4:**
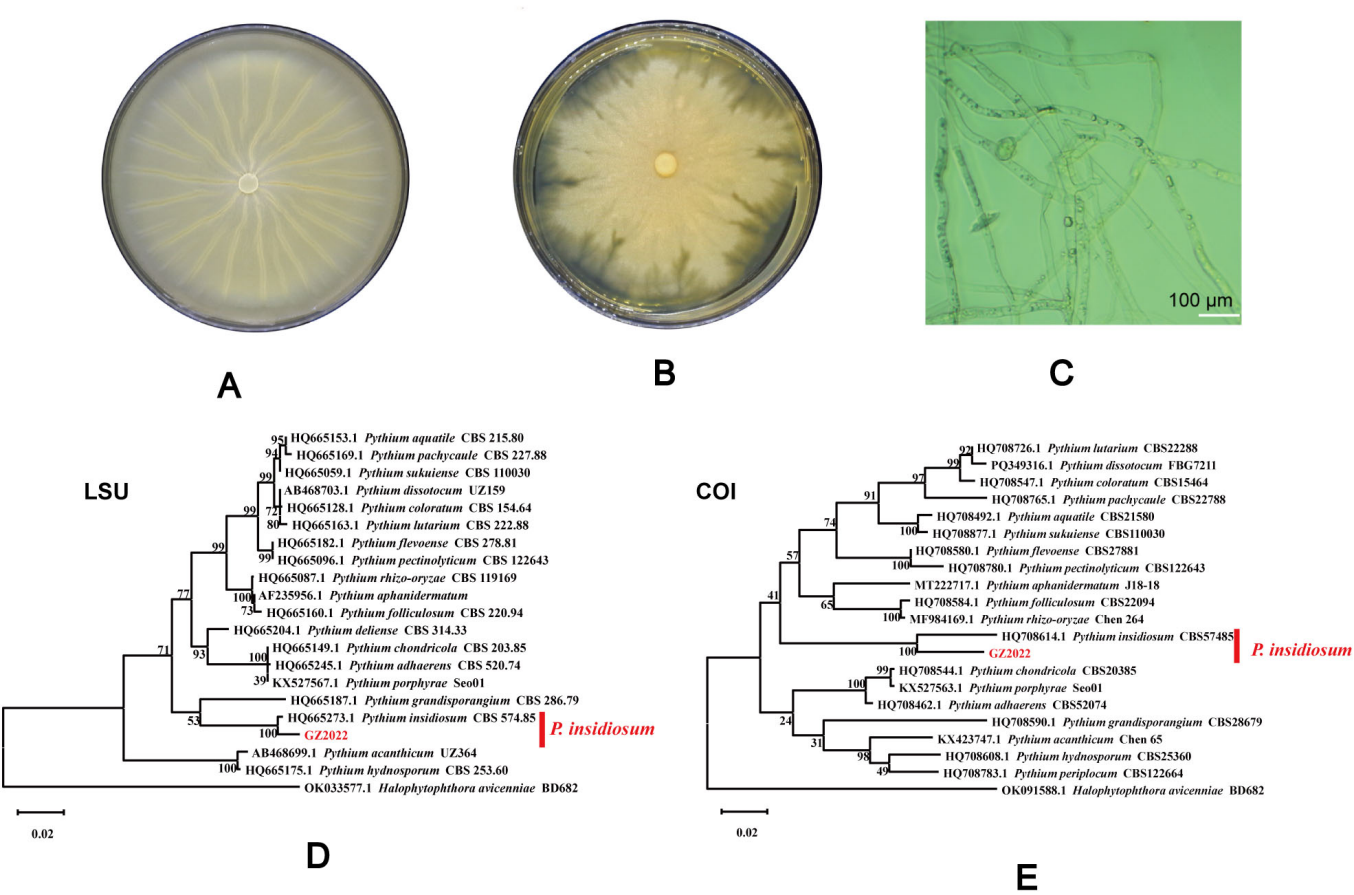
Culture morphology in different mediums and sequence construction cluster analysis tree of *P. insidiosum*. Culture morphology on brain heart infusion agar, and sabouraud dextrose agar **(A,B)**. Microscopic observation of the hyphae morphology of *P. insidiosum* **(C)**. LSU **(D)** and COI **(E)** sequence showed that the isolated strain was on the same branch as *P. insidiosum* CBS 574.85.

**FIGURE 5:**
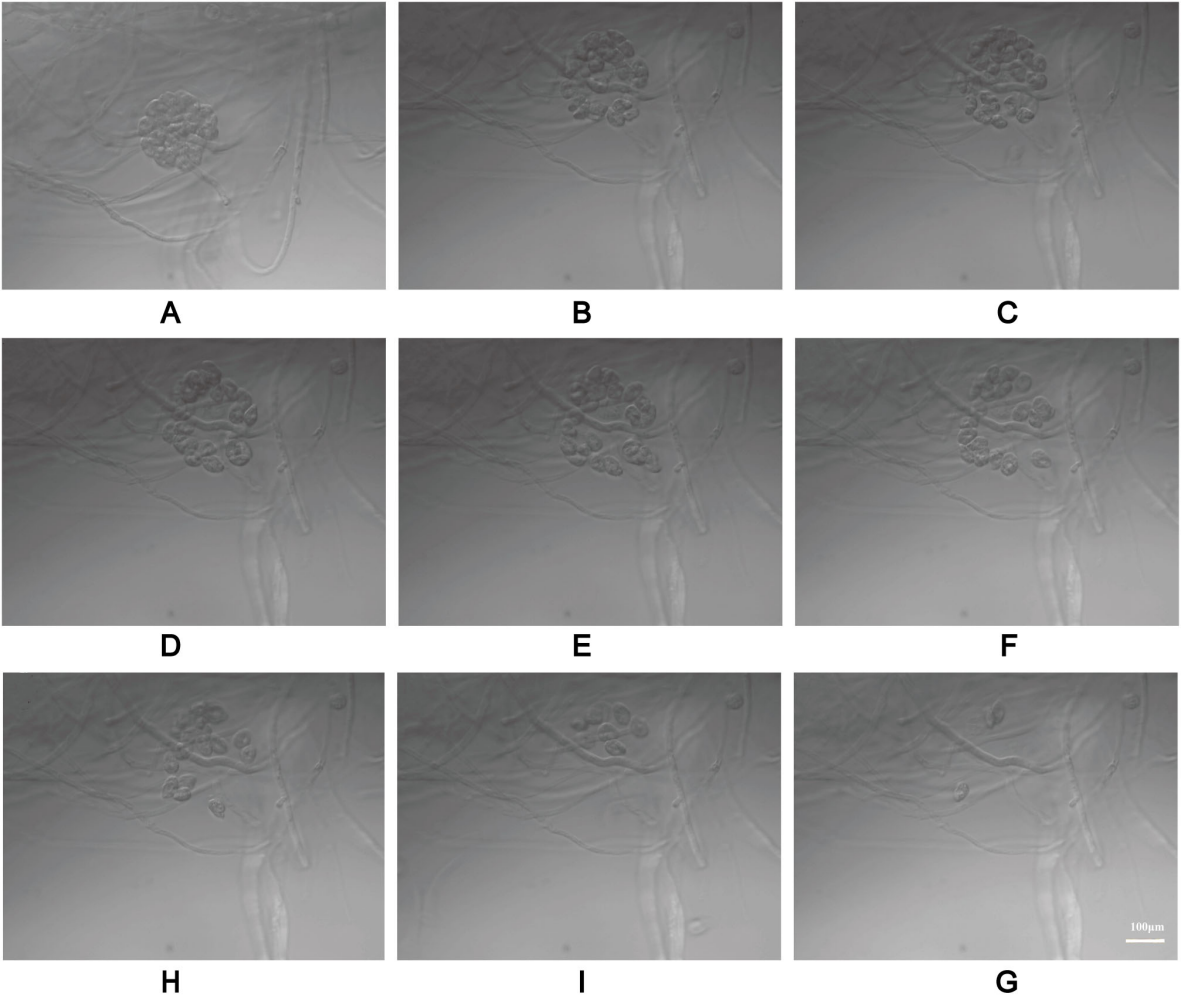
The process of zoospore release from *P. insidiosum* vesicles. The electron photographs showed globose vesicles leading to the release of secondary-type zoospores **(A–I)**.

**FIGURE 6:**
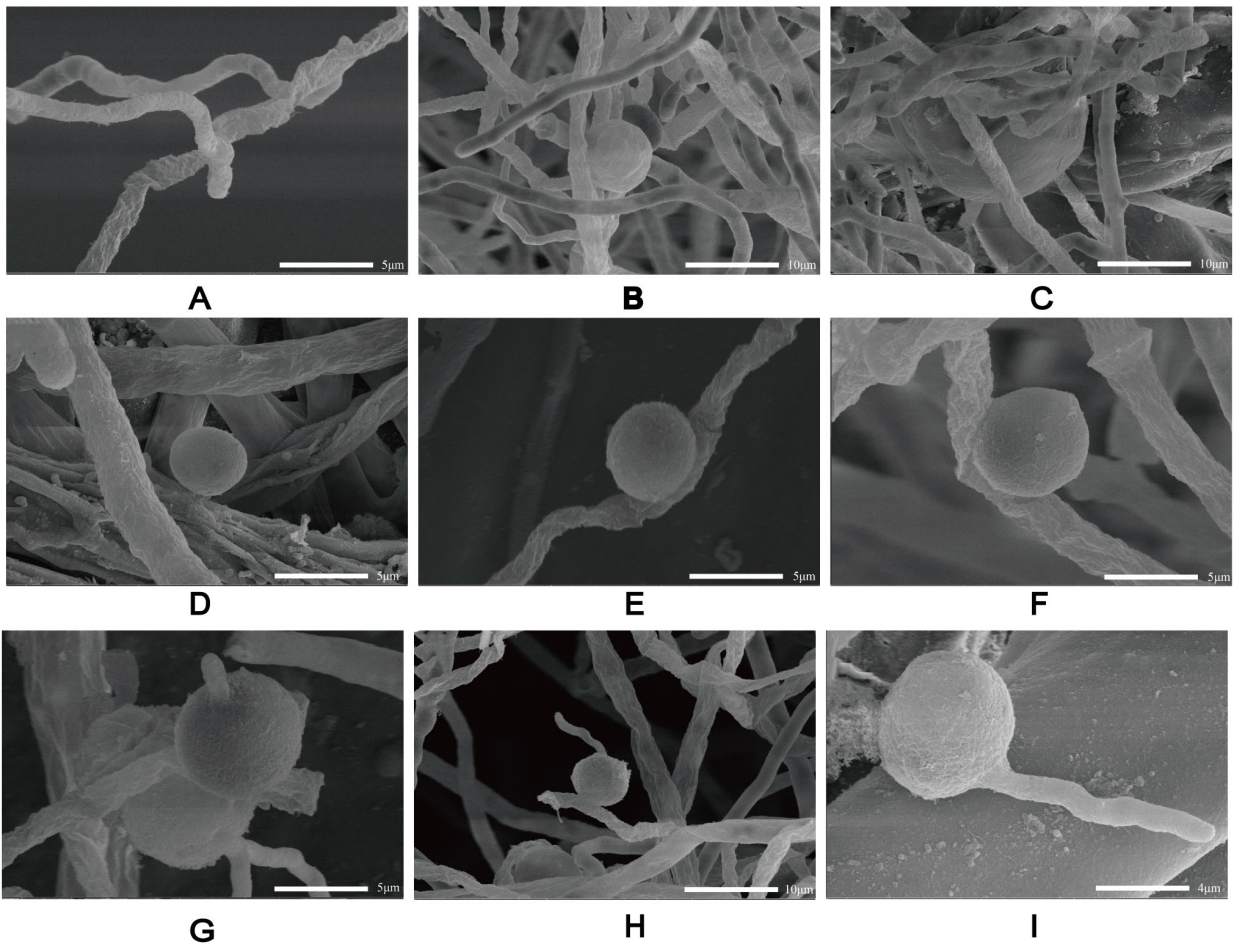
Scanning electron microscopy of *P. insidiosum at* different stages of development. Hyphae **(A)**. Sporangium **(B)**. Globose vesicles **(C)**. Spores **(D–F)**. Zoospores germinate into hyphae **(G–I)**.

Based on its microscopic features and phylogenetic analysis, the isolate was identified as a Pythium species. At 37 °C, *P. insidiosum* was characterized by white, sunflower-shaped, radiating colonies on brain heart infusion agar (Figure 4A) and submerged, white to colorless colonies with an irregular radiate pattern on Sabouraud agar (Figure 4B). The hyphae of *P. insidiosum* had lateral branches with diameters ranging from 4 to 10 μm. Older hyphae usually showed transverse septa, with protoplasmic nutrients flowing inside the hyphae (Figure 4C). Next, we conducted zoospore reproduction and SEM and TEM analyses of *P. insidiosum*. Zoospore release can be observed through microscopy (Figures 5, 6; Supplementary Video 1), and the process took about 35 min. The zoospores broke the vesicle wall and swam for approximately 20 min, eventually losing their flagella when they became resting spores (Figures 6D–F). When external conditions are suitable, the zoospores re-sprout to form hyphae (Figures 6G–I). In addition, the flow of protoplasm within the hypha was found (Supplementary Video 2). Traditional sporulation methods yield a small number of spores that are difficult to recover. In contrast, the improved method produced more spores that could be easily recovered and utilized. The spore yield of our improved method was 250 times that of the traditional method in shorter periods (Figures 1B–D; Supplementary Data Sheet 2).

The mitochondrial gene COI and the nuclear ribosomal gene LSU are frequently applied in phylogenetic analysis, species identification, and the exploration of evolutionary history. According to the BLAST results from the NCBI database, the COI and LSU gene amplification primer sequences shared a 100.00% sequence identity with *P. insidiosum* CBS 574.85 (Figure 4). The sequences of *P. insidiosum* were obtained through homology searches in the GenBank database. Phylogenetic analysis indicated that *the P. insidiosum* isolate in this study was genetically closely related to *the P. insidiosum* isolate from Costa Rica.

### 3.3 The inner structure of *P. insidiosum* with TEM

**FIGURE 7:**
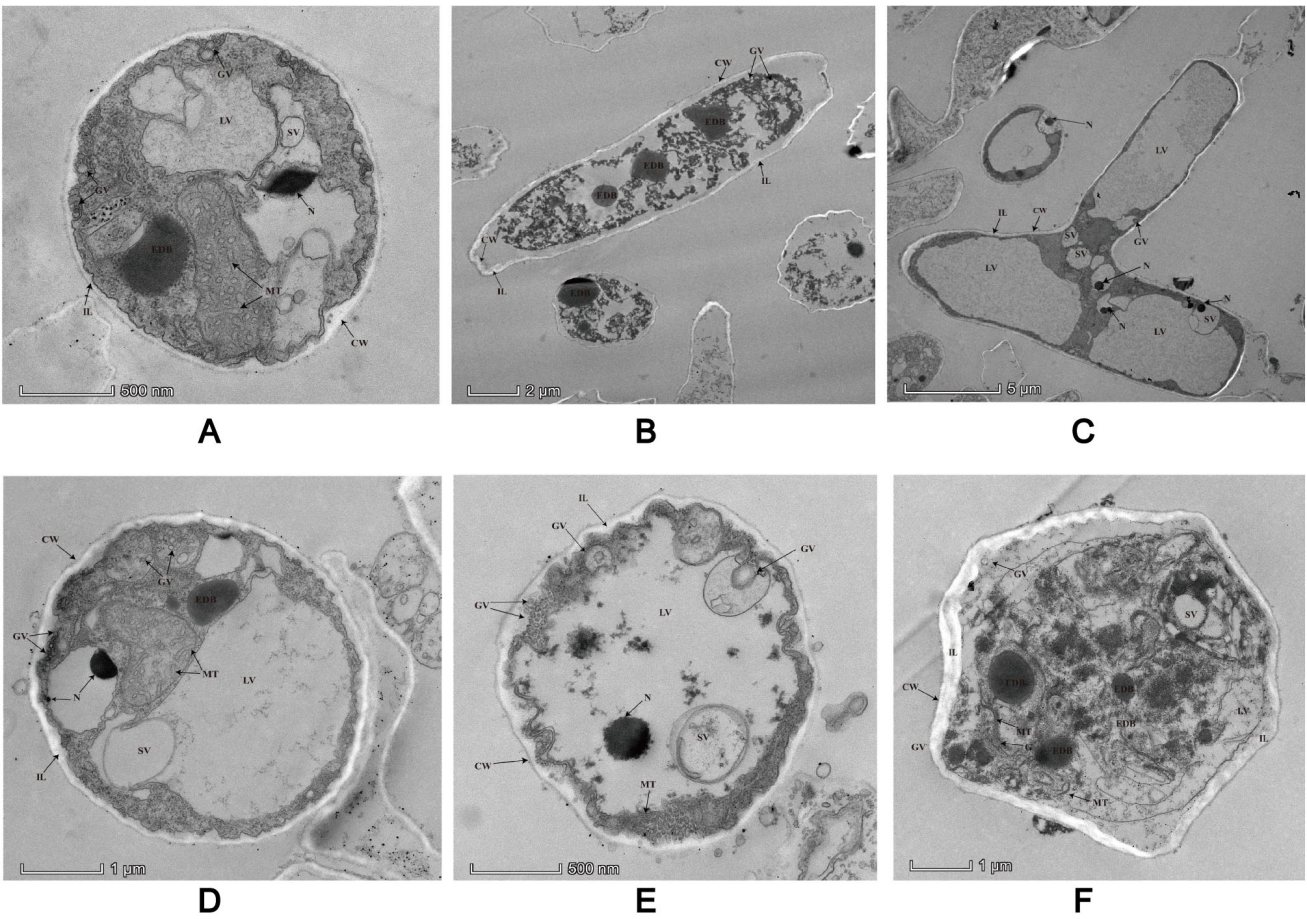
Details low magnification views of two different laterally sectioned *P. insidiosum* under TEM. All the structures are marked with arrows in the image. CW, cell walls; EDB, electron-dense black body; ER, endoplasmic reticulum; G, Golgi apparatus; GV, Golgi vesicles; IL, inner layers; LV, large vesicles; MT, microtubules; N, nuclei; R, ribosomes; SV, small vesicles.

*P. insidiosum* has a distinct cell wall consisting of several inner layers and typical internal organelles. The cell wall has a smooth plasma membrane (cytoplasmic membrane) associated with interlaced and meshed inner layers that form coarse and thick inner layers (Figure 7). Cytoplasmic vesicles are transported from the cytosolic compartment to the plasma membrane and integrated into the inner layer. Once integrated, the cytoplasmic bilayer membrane maintains its structural integrity. Large vacuoles containing black electron-dense bodies (EDBs) were observed within the cytosolic compartment. Some fields of the microscope of our sample showed nuclei, each surrounded by a bilayer membrane and containing granular chromatin (Figures 7A,D). Golgi structures with numerous Golgi vesicles pinching off from the Golgi apparatus were also detected in the hyphae. Golgi vesicles were found to integrate into the plasmalemma surrounding the cell wall. There were relatively large microtubules dispersed throughout the hyphae. Endoplasmic reticula (ER) were sometimes detected near the mitochondria, with multiple ribosomes around the ER. Numerous small vacuoles with fine granular contents, which varied in size and shape, were dispersed throughout the hyphae. Some stained samples contained numerous mitochondria with tubular cristae.

### 3.4 Drug sensitivity *in vitro*

To evaluate the effectiveness of existing antibiotics and antifungals against *P. insidiosum*, we conducted drug sensitivity tests *in vitro*. Our results showed that *P. insidiosum* was more sensitive to antibiotics than to antifungal agents. The MIC values of antibiotics against *P. insidiosum* were significantly lower than those of the antifungals (Table 1). Notably, DOX and AZM exhibited effective results against *P. insidiosum*. The MIC values for DOX and AZM were 4 μg/mL and 8 μg/mL, respectively. Other antibiotics, such as TIG, TET, CHL, and LZD, also showed varying effectiveness. In contrast, antifungal drugs such as fluorocytosine, amphotericin B, itraconazole, and fluconazole had much higher MIC values (higher than 128 ug/mL, indicating lower effectiveness. However, SMZ/TMP had a MIC value of 375 μg/ml. These results highlight the potential of using antibiotics, particularly DOX and AZM, to treat the infections caused by *P. insidiosum*.

**TABLE 1:** Susceptibility results for antimicrobial drugs against *P. insidiosum in vitro.*

Antimicrobial class	Drug	MIC (μg/mL)
Antifungal drugs	Fluorocytosine	256
	Amphotericin B	128
	Itraconazole	> 256
	Fluconazole	256
Antibiotic	Doxycycline (DOX)	4
	Tigecycline (TIG)	2
	Tetracycline (TET)	4
	Chloramphenicol (CHL)	16
	Linezolid (LZD)	16
	Erythromycin (ERY)	8
	Azithromycin (AZM)	8
	Gentamicin (GEN)	> 256
	Florfenicol (FFC)	4
	SMZ/TMP	375

### 3.5 Drug sensitivity *in vivo*

To certify the drug sensitivity results *in vitro*, we conducted the drug sensitivity *in vivo*. The mice in the PI group appeared to have coarse and disheveled fur, were listless, and had a dull response to stimulation after inoculation with zoospores 48 h. In contrast, those in the AZM and DOX groups appeared to have a more lustrous coat, were more vigorous, and were more alert to external stimuli during the entire observation period. One week after inoculation with zoospores, surviving mice in all groups exhibited hair loss and skin ulceration at the injection sites. Ulcerated lesions were observed at the inoculation site in mice infected with *P. insidiosum* (Figure 2B). Forty eight hours after inoculation with zoospores, the PI group showed mortality, while no deaths were observed in the control, AZM, and DOX groups. All deaths occurred within the first 5 days in the PI group, whereas the fewer deaths in the DOX and AZM groups were delayed 3–9 days. AZM and DOX treatments were more effective in promoting wound healing than the PI group (Figure 2B). The control and PI groups exhibited survival rates of 100 and 20%, respectively. In contrast, the AZM and DOX treatment groups had 80 and 90% survival rates, respectively. AZM and DOX significantly reduced mortality compared to the saline group, the AZM group showing a 3-fold reduction and the DOX group showing a 3.5-fold reduction in mortality (*P* = 0.0056 for AZM, *P* = 0.0008 for DOX) (Figure 2C; Supplementary Data Sheet 3). Next, to further confirm the drug effects *in vivo*, we conducted a detection of *P. insidiosum* load in the kidneys, spleen, liver, and lungs of BALB/c mice (AZM, DOX, PI groups) that were subcutaneously infected with *P. insidiosum* (Figure 2E). Microbiological organ cultures revealed that 80% of the infected mice in the PI-treated group had established *P. insidiosum* infections in the liver. In the PI group, Histological H&E staining revealed prominent neutrophilic periarteritis with thrombosis and perivascular inflammatory infiltration in the liver, lung, spleen, and kidney of mice (Figure 2D). In contrast, the histological features of the mice in the AZM and DOX treatment groups were improved compared to those in the PI group, indicating the therapeutic effects of AZM and DOX on pythiosis. GMS staining revealed fewer positively stained *P. insidiosum* cells in the tissues off mice in the AZM and DOX treatment groups. GMS staining of the PI group demonstrated a higher presence of black-dyed hyphae in the liver, while no hyphae were observed in other organs (Figure 2E). In contrast, GMS staining of tissues from mice treated with AZM and DOX showed no black-dyed hyphae, suggesting a significant reduction in *P. insidiosum* burden. Thus, treatment with the antibiotics AZM and DOX resulted in marked improvement in histological lesions and a substantial decrease in *P. insidiosum* load *in vivo*.

## 4 Discussion

Pythiosis is a severe disease with high morbidity and mortality, particularly in patients with disseminated infections or vascular diseases (6). The recent reports of pythiosis in humans are gradually increasing and require more attention. Our epidemic distribution map data reaffirmed Asia’s disproportionate pythiosis burden, with 94.83% of global cases concentrated in India and Thailand. Notably, China’s 1.75% case contribution calls for heightened vigilance. Frontline clinicians in ophthalmology, dermatology, and infectious diseases must pay more attention to pythiosis, which has been reported in 17 countries. The systematic synthesis of historical case data distribution enables healthcare providers to rapidly assimilate critical diagnostic biomarkers and effective intervention strategies for suspected cases, thereby reducing the time-to-diagnosis and improving the survival rate.

The cultivation-based approach remains one of the most important methods and provides additional advantages for susceptibility testing for *P. insidiosum*. Identifying *P. insidiosum* based on morphology alone is challenging and insufficient because of the restricted morphological differentiation in culture (32). Direct sample multiplex-PCR, a potential tool for rapid equine pythiosis diagnosis, overcomes morphological identification limitations and provides a definitive diagnosis (33). Molecular phylogenetic analysis is a reliable method for identifying *P. insidiosum* using COX and LSU (12, 34). Our results suggest that the isolate is more closely related to *P. insidiosum* than to any other Pythium species, indicating that it is the same species. This improves the accuracy and dependability of our subsequent findings. The novel sporulation protocol developed here addresses longstanding cultivation challenges based on the improvements made in earlier studies (24). Although molecular assays are increasingly employed, conventional culture followed by zoospore induction is still regarded as the gold standard (14). We combined an induction liquid and Annona Squamosa L. leaf extract, which significantly increased zoospore production compared to existing techniques. This was a critical advancement in the research on the biological characteristics of *P. insidiosum*. Traditional methods often involve complex and time-consuming procedures. The traditional method requires nearly 27 h to induce enough zoospores, while ours requires 10–24 h, and the number of zoospores produced using our modified method was 250 times that of the traditional method. Our modified method streamlines these steps, making the process more efficient and accessible to researchers. Our modified method may be a step forward in microbiology, specifically in oomycetes, and potentially accelerate the development of new treatments or preventive measures against pythiosis. In conclusion, the modified method for inducing sporulation in *P. insidiosum* was more efficient, time-saving, and simplified than the traditional methods.

To better understand the life cycle and the internal morphological and structural characteristics of *P. insidiosum*, we used TEM and SEM to elucidate the dynamic changes in *P. insidiosum* from hyphae to zoospores and examine its internal cellular structure with TEM. Our observations reveal several key ultrastructural features of *P. insidiosum* hyphae and zoospore, including the cell wall, inner layers, vesicular transport system, and other organelles. These detailed structural insights provided a foundation for understanding the organization and function of *P. insidiosum* at the cellular level. The detailed visualization of *P. insidiosum’*s cellular structures via TEM, including its cell wall and antigen distribution mapped through immuno-electron microscopy, provides a foundation for understanding its pathogenesis and suggests potential targets for diagnostic and therapeutic interventions (27, 35, 36). Therefore, understanding these structural features is essential for identifying potential targets for developing new antimicrobial agents.

The treatment of pythiosis has seen considerable advancements; however, it remains a complex challenge. Tetracyclines, macrolides, aminoglycosides, and other antibiotics (such as LZD, nitrofurantoin, quinupristin-dalfopristin, CHL, clindamycin, and mupirocin) have demonstrated inhibitory activity against *P. insidiosum in vitro* (20). Our results *in vitro* showed that *P. insidiosum* was more susceptible to antibiotics than antifungal medications. This conclusion is similar to the previous study, which found that antibiotics inhibit *P. insidiosum* 100 times more than antifungal agents (37). Tetracyclines and macrolides may also be potential candidates for infection control, consistent with these studies (37, 38). *In vitro* drug sensitivity tests showed that sulfamethoxazole had no bacteriostatic effect. However, the patient with the isolated strain of P. insidiosum was cured with a multi-drug plan containing SMZ/TMP and antifungal drugs, with SMZ/TMP as the only drug at the end. This efficacy discrepancy between *in vitro* and *in vivo* is not uncommon and can be attributed to several factors. Firstly, the metabolic state of *P. insidiosum* can change *in vivo*, which may influence its susceptibility to drugs (39). *P. insidiosum* can develop mutations of the dihydropteroate synthase gene *in vivo*, which is the target of SMZ/TMP, and change the drug sensitivity. Secondly, SMZ/TMP has been shown to trigger allergic reactions and enhance immune function which inhibit the growth of melanoma skin cancer (40). Therefore, it is plausible that SMZ/TMP may enhance the host immune system to exhibit anti-*P. insidiosum* effect. However, further investigation is needed to validate these mechanisms.

Drug sensitivity experiments conducted *in vivo* have indicated that DOX and AZM are the main antibiotics used to treat pythiosis (41). Our mouse model successfully mimicked vascular/spread pythiosis in humans, demonstrating systemic illness following subcutaneous injection of *P. insidiosum* (25). Investigations *in vivo* corroborating our *in vitro* findings indicated that AZM and DOX alone showed positive therapeutic effects in the treatment of pythiosis in mice. Both medications significantly improved histological changes and survival rates (*P* < 0.05) and reduced the fungal load in the liver, echoing the results of previous studies (42–44). Our results further confirm that mice are a reliable model for *P. insidiosum* infection, and we recommend their use in future studies. However, our study was limited to a single *P. insidiosum* strain, and the mechanisms of action of AZM and DOX against *P. insidiosum* remain elusive, possibly involving the inhibition of protein synthesis, cell wall synthesis, and/or amino acid transport (43). In China, where infection rates are low but increasing, it is essential to analyze data on this strain thoroughly to develop effective treatments for the disease.

## 5 Conclusion

Epidemiological investigations have revealed that India and Thailand are hotspots for human pythiosis, and sporadic cases are increasing in China, the Americas, and Europe. We performed comprehensive tests, including molecular biology, morphology, and biological processes, to confirm that this strain was *P. insidiosum*. We developed a modified spore-producing method and showed the microstructures with TEM, which may benefit future studies on *P. insidiosum*. Finally, through drug susceptibility testing *in vitro* and *in vivo*, we successfully reconfirmed effective drugs for pythiosis, which will provide theoretical and technical support for the rapid diagnosis and treatment of outbreaks of such diseases in inexperienced areas with few epidemics. We recommend that AZM and DOX be considered in future *in vivo* experimental studies evaluating the treatment of pythiosis.

## 6 Limitations of the study

The epidemic distribution depends on the reported data, which would not be very accurate for some cases that are not reported. The limited number of isolates tested and the specific dosages and durations required for drug combination experiments have not yet been explored. We have also not conducted further studies on the mechanism of its therapeutic effect. Thus, further research, including a more significant number of isolates, especially from genetically diverse *P. insidiosum* strains, different drug regimen treatments, and associations with other antimicrobials, immunotherapies, and surgery, will provide a better understanding of the therapeutic potential of AZM and DOX in pythiosis treatment, as well as the pathogenesis of *P. insidiosum* infection in humans and animals. Our efforts should also include elucidating the exact mechanisms of effective sulfanilamide in our patients.

## Data Availability

The original contributions presented in the study are included in the article/Supplementary material, further inquiries can be directed to the corresponding author/s.

## Footnotes

1
https://pubmed.ncbi.nlm.nih.gov

2
https://scholar.google.com

3
https://blast.ncbi.nlm.nih.gov/Blast.cgi
