# Supplementary material for: Worldwide distribution of human pythosis and biological characteristics of a Pythium insidiosum strain susceptible to antibiotics from China: a dual-scale study
Source: Front Med (Lausanne). 2025 Dec 9;12:1629018. doi: 10.3389/fmed.2025.1629018 (PMC12722454; doi:10.3389/fmed.2025.1629018)
Supplement: Supplementary file 1 [file Image_1.pdf]

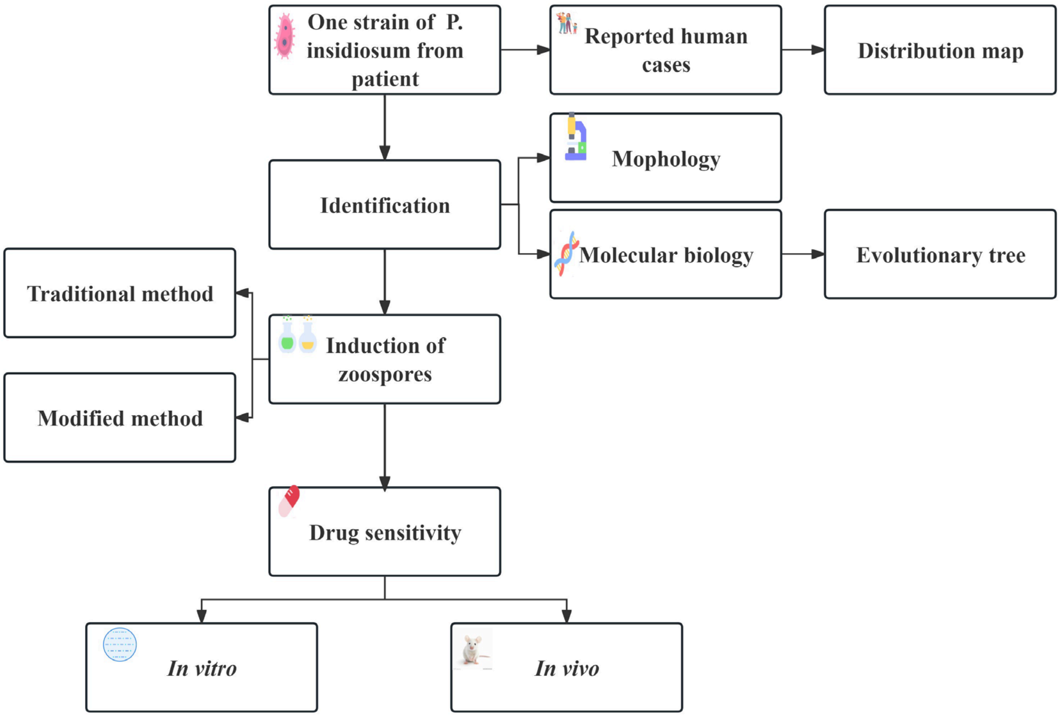

Fig.S1 Flowchart of the study design.

Video S1 The process of the zoospores production under the microgram.

Video S2 The flow of protoplasm within the hypha under the microgram.
